# Address before the Medical Society of the State of New York

**Published:** 1870-03

**Authors:** Jales P. White

**Affiliations:** Buffalo, President


BUFFALO
Medical and Surgical Journal.

VOL. IX. MARCH, 1870. No. 8.

### Original Communications.

ART. I.—*Address before the Medical Society of the State of New York.* By JALES P. WHITE, M. D., *of Buffalo, President. Delivered February 2d.,* 1870.

(Reported for the Buffalo Medical and Surgical Journal.)

FELLOW-MEMBERS OF THE SOCIETY, LADIES AND GENTLEMEN :

In conformity with a by-law of this Society, and following the example of my distinguished predecessors, it becomes my duty to address you on some subject which may be expected to interest both the members and the lay friends of the profession who honor us by their presence. The number of topics from which selection can be made for an occasion like the present is somewhat limited, being confined to such as are not exclusively scientific in character, and which occupy our business sessions, but combine a popular element, adapted to the entertainment of our invited non-professional guests. Hoping to avoid the charge of professional egotism which the hyper-sensitive may think incurred, your attention is invited to the subject of *Progress in Medicine.*

There exists a popular prejudice that Medical Science is stationary. Irregulars of every stripe foster this opinion, and assert that the profession is non-progressive. Indeed by all the " isms " which infest society, is legitimate medicine charged with being " fossilized." Against these and similar accusations, we shall attempt to vindicate that profession, to the prosecution of which our lives are devoted. We might content ourselves by demanding of those who make this groundless assertion against the true disciples, to point out a single instance in which progress has been accomplished by one of these presumptuous revilers. What Physiological law ? What

Pathological truth? What Chemical discovery? What rational Thærapeutical combination? What important Thærapeutic principle can be claimed for those who thus assail the true Esculapian ranks? We might be satisfied to dismiss the further consideration of the charge against "progressive medicine" until its enemies shall establish a character for their own achievements; yet does the observation of one whose acquaintance with the profession extends over a period of more than forty years, induce the belief that we may safely undertake much more. A cursory glance at the advancement made in the several departments of Medical Science within the experience of an individual who hopes yet to see much more accomplished, will clearly demonstrate that it can claim a degree of progress unsurpassed in any other field of human effort. As when the broad Niagara, emerging from the Lake, and long before it takes the fearful plunge, from its great depth remains undisturbed by the pebbles below, and rushes noiselessly on, and only by fixing the eye on some point on the opposite shore, or some object floating upon its surface does the beholder become aware of the rapidity and force of its current; so with the unobtrusive advancement of medical science, its votaries can only realize the giant strides which are constantly being made, by carefully observing its condition in the past, and noting the changes which have taken place within a limited period. Let us therefore summon the several departments before us, and allow each an opportunity for self vindication. What forward steps have they taken during the last two score years, as observed by those of us who have been borne along upon the current of that period.

In proceeding to a seriatim cross-examination of the several subjects forming the composite called "Medical Science," descriptive Anatomy, one of its rudimental constituents may be first cited to trial. Anatomy is not only the basis of Physiology but of Pathology, and the practice of Medicine. It is therefore proper that we should commence this investigation by ascertaining what advance has been made in a knowledge of the structure of the human body. The ignorance and superstition which among the intellectual Greeks, and war-like Romans had prevented dissections, had at length been overcome. The proper study of Anatomy, which had been encouraged upon the continent was finally legalized in Great

Britain in the second year of the reign of William the IV, by a Parliamentary enactment, permitting the possession of human bodies for the purpose of dissection. In passing, we may remark that this judicious enactment in the British dominions, like similar legislation in all parts of the world, was sufficient to arrest the crime of body-snatching, and the still more revolting one of Burkeing. It also brought into this field a large army of active men, whose careful dissections and investigations left no part of the human system undisclosed. Indeed, at the commencement of the period now under consideration, and when the speaker entered upon the study of this department, in simple descriptive anatomy of the parts of the human system, it is but truth to admit that few deficiencies were left to be supplied. Doubtless the recent works of Wilson and Gray with their numerous illustrations facilitate the acquisition of the knowledge of the science, yet the authors which were in the hands of the student of that period such as Bell, Cloquet and Wistar, would not be inappropriate as text books for the student of to-day, and a large atlas of plates of the bones, muscles and organs, published in 1821, by John Lizars, of Edinburgh, and now in my possession, cannot be surpassed for scrupulous correctness and perfection of outline, by any of its more pretentious modern successors. The now venerable Prof. James McNaughton, of this city, to whose lectures on Anatomy it was my privilege to listen, in the years of 1831–2–3, described with unsurpassed accuracy the minutest points of origin and insertion of every muscle, the situation and form of all the viscera, and the smallest process of every bone entering into the formation of the human body. Granville Sharp Pattison, then of Philadelphia, whose brilliant course upon the same subject I subsequently attended, had made the great advance of carefully describing the organs and their relations to each other in the regions most frequently involved in surgery, or regional anatomy. Bichat had also recently called attention to the anatomy of tissues, and had pointed out its pathological importance. Here, this the most advanced of all the departments, rested. Minute anatomy remained an unexplored field. The Microscope had hardly been called in requisition. Microscopical anatomy of the tissues and fluids was unknown, and the analysis of the solids and fluids, or animal chemistry, remained nearly a sealed book. The marble had been quarried, and had received certain outlines and proportions, but it required progress in the associate departments of Physiology, and Pathology to inspire it with vitality. It was dead, inert, unfinished matter, minutely describ ed it is true, but requiring that inspiration which it derives from the study of its healthy functions, and Pathological changes, to give it life, and utilize it for the medical practitioner. The science of organization remained comparatively useless—a mere arbitrary collection of names, without any application of the science of life or Physiology, or any knowledge of the changes occasioned by disease or Pathology. These departments were as yet, almost entirely undeveloped.

Let us next then inquire into the state of the science of Physiology at that period. Adelon, Chaussiere, Blumenback, Richerand, Magendie, Rudolphie, Broussais, Sir Charles Bell, and others had by their writings and experiments been instrumental in creating a taste for the prosecution of physiological studies. It is however an undoubted fact, that nearly all important discoveries in physiology have been the result of experiments upon living animals—called experimental physiology. This method of investigation was scarcely practiced in any part of Europe at the time now under consideration, and had no existence in this country.

Under the lead of such men as Robin, Lebert, Bernard, Coste and Longet in Paris, and Dalton and Flint in America, has experimental physiology advanced the science with wonderful rapidity, and uprooted many of the theories taught when I became a student of medicine. Much has been demonstrated, the existence of which was then unsuspected. So numerous and important are these changes that physiology may almost be said to have been recreated. Illustrations in proof of this assertion are so numerous as scarcely to permit selection. Take for instance the subject of generation. Drelincourt in a curious and exhaustive work on this subject enumerates no less then 263 theories which had been described, and many more might have been added. Several of these theories were taught and believed by different physiologists when the speaker first sought instruction on the subject. These with their predecessors are now demonstrated to be incorrect and are regarded as little if any better than the speculations of Aristotle and Hippocrates, which had been taught more than two thousand years before. This seems to have been the favorite study of the great Harvey, and after the discovery of the circulation of the blood he made many observations worthy of that sagacity and industry which were never exceeded. With all his abilities and advantages, and when it was reasonable to expect that he would be silent or say something satisfactory on the subject; he contented himself with his incomprehensible "Magnetic theory." Our own countryman, Dr. Dewees entered this fruitful field of speculation with the same fruitless results. In this hypothetical and unsettled condition was the physiology, of these functions when I entered upon its study. In 1842 M. *Pouchet, Professor of Zoology in Rouen, first published his work entitled "Theorie Positive de la Fecundation," in which he announced the discovery of the ovular theory.

Negrier, Patterson, Lee and Jones seem to have been on the very verge of this discovery, and were apparently prevented from making it by too rigid adherence to old theories. Pouchet announced the law that generation in all animals takes place by means of ova or eggs; some inferior beings alone forming an exception. He established the pre-existence of ovules in the whole animal series, without the possibility of question, and their periodic emission independently of fecundation or the influence of the male germ. Thus did he assume to overturn all previous opinions relating to the functions of the ovary. His theories have been sustained and fortified by direct experiments made by Bischoff and Raciborski as well as by himself and by all there at nearly the same time and independently of each other. Subsequently M. Coste and many others in France, and Dalton, in this country, have confirmed these position and the doctrine is now believed to be firmly established. Again, until within the time now under consideration it was believed that animals and plants differed very widely—that their substance had nothing similar and that cells existed only in plants—such was the condition of things when Schwann, of Germany, taking up the beautiful investigations of Schlieden, published in 1838, upon the structure and growth of vegetable cells; came to the conclusion that animal tissues con-

* I have a copy of his first Monograph given me by the author in 1851, which, in my opinion, establishes his right to priority.

sisted equally of *cells*, and that whatever may be the complication of this substance in the animal—whatever may be the external form of the various parts in the animal tissues—they all originate from *cells*, and are after all only modified cells. We find also that in the beginning the germ consists of simple cells, derived from a modification of the *yolk* which is an essential part of all ova or eggs. Such then is the condition of all germs, and from this starting point we arrive at all animals, even so complicated in structure as man. In other animals, throughout the series of the animal kingdom, in which the most complicated structures are observed—in which structures very distinct and unlike are successively formed, flesh, blood, nerves, skin, hairs, scales and all possible structures, so unlike as to admit of no comparison, all are now known to be formed in the same manner. They are merely modifications of the cellular tissue which characterized the germ when forming. What we call ova or eggs then, in their simple condition, are but cells of a peculiar structure, formed in a peculiar part of the body, —destined to undergo peculiar modifications, by which the body is not enlarged, by which no particular function is performed, but by which a new individual is created. Thus do the investigations of modern physiologists establish *unity* in the *structure* of animals and rarity in their mode of reproduction. The time is now past when it is possible to doubt that there is order in nature—the existence of a general law regulating the creation and development of all animated beings cannot be questioned.

These discoveries alone established, as they have been within the period of my own observation, would be sufficient to establish the progressive state of physiology, and entitle us on this evidence alone to a triumphant vindication.—This, however, is but a single advance and many others equally important may be mentioned. Sir Charles Bell, or rather Magendie, had just described the spinal marrow and demonstrated the existence of sentient and motor filaments and this was then regarded as a large step forward. But Sir Charles Bell was utterly ignorant of the function of spinal reflex action and the important role it plays in the production of disease. How much has been learned also during the last forty years of the functions of ganglionic or organic nerves, the important part they play in digestion, nutrition, assimilation, circulation and respiration. Who at the commencement of the period now under consideration had any just appreciation of the functions of the liver, although, through ignorance of its condition most patients were assurred that they were *bilious* and severely medicated upon the hypothesis. In 1849 M. Claude Bernard first described an important function of the organ, till then unsuspected under the name of the "glycogenic function of the liver." The experiments of Bernard, the most important of which have been repeatedly confirmed by other observers, demonstrate that, most of the sugar so formed has an internal origin, and that it first makes its appearance in the tissue of the liver itself. During the year 1869, G. F. Barker, M. D., has published a series of articles in Silliman's Journal, confirmatory of the theory of the formation of sugar in the liver, and supplied abundant additional proof of its correctness.

The discovery of the excretory function of the liver, in 1862, is one of the most important of modern times. Prior to this date the existence of cholesterine had been demonstrated in various parts of the economy, but its physiological history was completely unknown. By a series of elaborate experiments Prof. Austin Flint, Jr., conclusively established that this substance is formed in the brain, and nervous tissue by destructive assimilation,—taken up from these localities with the blood, conveyed to the liver, and in this organ separated from the blood, and as an ingredient of the bile, discharged into the intestine. The existence of a depurative function of the liver, equal in importance to that of the kidney is thus clearly shown. The recent investigations of Bernard, upon the temperature, pressure and modifications in the circulation of the blood in the glandular system, the analysis of its free gases, together with the discovery of the function of the Pancreatic juice in the digestion of fats are all striking examples of the progress made in this department of medicine.

The views of physiologists with regard to the essential process of respiration and hematosis before the time of Lavoisier are completely exploded and now possess merely historical interest. Sir Humphrey Davy, in 1839, in an essay on "Light, Heat, and the combination of Light, with a new Theory of Respiration," first advanced the accepted doctrine of the present day. But enough has been said to fully establish the highly progressive state of physiology, and without dwelling longer in this most inviting and fairy field we must press on to a consideration of the other departments.

*Materia Medica and Therapeutics* being also rudimental, may next be summoned to show progress. "This Department," says the learned Prof. Charles A. Lee,* "is the most important of all branches of Medicine" all others are mainly subservient to it, and yet it has received less attention from the profession than any other in the *Curriculum* of Medical study, it has always been undervalued, both by teacher and student.* Although this opinion of its paramount importance may arise in part from the zeal of an enthusiastic teacher of this department; still all must admit that it is difficult of attainment and requires the most profound study. It implies a thorough knowledge of all medicines; their natural history; chemical composition; physiological and therapeutical effects, their *modus operandi* in all morbid conditions; effects in over-doses; mode of producing death, antidotes, and especially an acquaintance with all modifying causes; their indications and contra-indications; as well as modifications of effects produced by combinations; all this requires years of study, close observation and experience.

This department has been greatly advanced by a better acquaintance with pathology which exists now as compared with forty years ago. A successful application of remedies to disease must be based upon a knowledge of the seat and cause of the disease, as well as the *modus operandi* of medicines administered. The popular mind regards disease as occasioned by some poison, for which there must be some antidote,—a specific. All homœpathic remedies are regarded in this light by those who prescribe them. The true use of drugs has formerly been to a great degree *empyrical*—based upon much that may be called false experience, prescribing for symptoms and overlooking the cause, seat and origin of the disease. Again in judging of the influence of drugs in healing diseases, physicians are too much in the habit of overlooking the "*vis medicatrix naturae* or natural recuperative power; thus attributing to medicines what

---

*Introductory Lecture Delivered before his Class at the Buffalo Medical College, Nov. 25, 1869.

was properly due to the innate curative forces. The adoption of theories was among the causes retarding progress in therapeutics. The Brunonian, the Broussain, the Humoral, the Solidary, and many others might be mentioned, all of which required their advocates to accomodate their prescription to a foregone conclusion. Upon these points much more rational views are entertained by practitioners of to-day, than prevailed forty years since. The medical mind is less the slave of prejudice, and prescribing becomes much more the result of a rational analysis of the existing pathological condition and symptoms. Remedies are adapted to emergencies, irrespective of hobbies or theories. Who, at this time thinks it necessary to abstract blood freely from all patients without regard to their condition, when apoplexy is diagnosed? And yet when I entered the field of practice, criminal neglect would have been charged upon the man who had hesitated in the use of the lancet, no matter how anæmic the patient.

One of the principal facts showing the progress of knowledge in Therapeutics, and its cognate department, Hygiene, is the increased value of human life as shown by numerous mortuary tables, and by the constantly increasing profits of Life Insurance Companies. The diminished mortality from special diseases in civilized nations, the disappearance of epidemics and endemics, which but a short time since were vastly destructive to the human family, and the better control of those remaining, abundantly prove immense progress in Therapeutics and Hygiene. Great improvement has taken place in this department of Medical Science within a comparatively short period through the progress made in chemical knowledge. The isolation of the active principles of vegetables,—Quinine, Morphine, Strychnine, and all the family of alkaloids, has greatly contributed to advancement in therapeutics. Who, that had a case of intermittent to treat forty years ago does not shudder at the recollection of the horrible efforts necessary to "choke down" sufficient crude bark to arrest the paroxysms, and thank God that he can now prescribe, through modern improvement in this department, a far more efficient, compact, and acceptable dose. Not only have the active principles of many of the remedies been discovered and brought into use during the last two score years, but there has also been made a long list of new and valuable additions. To this class belong the whole family of Iodides and Bromides, as well as all the Vegetable Alkaloids, to which reference has already been made, and we may also add Cod Liver Oil, and a large number of plants indigenous to this country. The best antiseptics, deodorizers, disinfectants, the per-manganate of potash and carbolic acid—and the application to the same purpose, of the salts of iron, have all been introduced during the period under consideration. The increase in the number and the improvement in the combination and mode of administering iron, would alone entitle this department to triumphant vindication against the charge of non-progressiveness.

Permit me to call attention to the introduction of anæsthetics so recently discovered that the oldest child, whose birth was accomplished under its humane influences, has scarcely attained his majority. We might claim a verdict in favor of our client, and rest it solely upon our ability now to relieve pain. Kindred to the relief of suffering by anæsthesia, is the modern method of introducing medicines into the system by the hypodermic syringe, thus securing their effects with greater rapidity and certainty, and often when the stomach is incapable of receiving and retaining them. Among the improvements which have been made in this department, may be mentioned, and as among the most important, simplification of prescriptions—giving one or two reliable remedies for a specific purpose, instead of combining a multitude often incongruous and never rational. Immense changes for the better have taken place in pharmaceutical preparations, frequently rendering the remedy which formerly was nauseating and repulsive, acceptable to the most fastidious taste—of this class we may mention the extracts, the dragees or sugar coated pills, the syrups, the elixirs, conserves, capsules, etc., etc., of to-day, as compared with the clumsy and disgusting preparations of the same drugs, unavoidably prescribed a few years ago. The knowledge of the cause of scorbutus, and of the appropriate remedies for its prevention and cure, does not ante-date the period under consideration. The subject of alimentation in disease, has only within a few years, through the labors of Prof. Austin Flint and others, received that attention which its importance demands. Much has been accomplished by directing attention to this subject.

Formerly the sustentation and comfort of the patient was overlooked, and he was subjected to impure air, starvation, thirst and heat. Forgetting, in a vigorous attack upon the disease, with *heroic remedies,* to secure Nature's co-operation in effecting a cure.

The merits of the practitioner were estimated by the number and activity of the remedies which he brought to bear upon the enemy to be routed. All the circumstances which modify the effects of medicines are far better understood than formerly, as idiosyncrasy, sex, age, temperament, diathesis, habits and modes of life; race, passions and appetites, the state of the mind, season of the year, light, air, food, climate, temperature, etc., etc. But we cannot dwell longer upon this interesting department. Enough has been said to establish great progress in Materia Medica and Therapeutics, and we pass to a similar examination of the claims of Chemistry, another of the fundamental branches of the Science of Medicine.

Every science is slowly and gradually developed, and Chemistry forms no exception to this rule. It is still, I trust, in its infancy, but nearly all we now know of it, is the result of investigations and experiments during the last century.

The Alchemists did not study chemistry as a science. They practiced it as a means of attaining other objects. They searched for the Philosopher's stone, they essayed the conversion of the baser metals into gold and silver, and attempted the discovery of the elixir of life—a universal panacea. In this pursuit they failed, but their indefatigable industry, and their wonderful ingenuity, led to numerous discoveries, neglected at the time, in many instances, which have had a most marked influence on the development of modern science. It was not until 1760, or 1770, that Lavoisier first gave form to chemical studies and effected a complete revolution in the science. From that time chemistry has made rapid and continuous progress. The time had at length arrived, when the materials collected by the ancients with so much assiduity and zeal, were made the foundation, on the Baconian or inductive system, of a new science. A host of laborers, now eagerly rushed into the field. Chemical combinations, combination in definite proportions, the atomic theory, combination by volumes, atomic or equivalent volumes, chemical nomenclature, chemical analysis, and resolving, many supposed, simples into their elements, were soon brought to the attention of the student, and gave to the science form and certainty. Great advance had been made in this department before the time when our investigations commence, and its application to animal tissues and fluids in health and disease, and to the analysis of vegetable substances, had made some progress, but its full realization is but now beginning. Perhaps no more notable change has taken place than in the Synthetical experiments, and the results which have been accomplished therefrom, within a few years. Organic compounds can be artificially made out of their elements, or out of mineral compounds, and are not, as has been until recently supposed, exclusively the products of living beings. Latterly Synthetical chemistry has in no inconsiderable degree taken the place of analytical, and chemists have succeeded in building up many of the organic compounds contained in our bodies; there are those indeed who maintain that the day is not far distant when she will call into existence low forms of animal life.* Already various forms of albumen are claimed to have been produced, while there is literally no end to the number of quinary and quaternary compounds that chemistry has produced, and is yet producing. Perhaps the greatest and most important truth disclosed in the progress of organic chemistry is this,—whatever compounds we have containing one atom of carbon, and whatever may be its properties or its relations to other bodies—we find all these repeated in compounds containing two atoms of carbon, and again in those containing three atoms, and so on. Hence we have the relations termed homologous and heterologous, and organic bodies are to be studied in two entirely diverse directions. It is a consequence of these new established principles that, although the compounds of carbon now known and described are almost innumerable, yet organic chemistry is easier of acquirement than it was a few years since, when all its known bodies could be easily enumerated. Nor has analytical chemistry been neglected. Its usefulness has been extended to the arts. For example, a few years since the black, offensive tar of our gas works was thrown away—entirely refuse—worse than refuse, a positive nuisance—hard to be rid of. Now we extract from it substances convertible by the art of the chemist into a complete series of colors—rivalling or sur-

*Mr. S. M. Bradley, Introductory Lecture, delivered to his class at the commencement of the session of 1869–70, in the Manchester Royal School of Medicine, England.

passing the most brilliant of the vegetable or animal world. To the same unpromising source, we owe another substance of great value, and perhaps still greater promise in medicine—the Phenyl alcohol, or carbolic acid. Time would fail me, were I to attempt, to give even a list of the new compounds discovered and prepared by chemists in the last few years; some of them of great scientific interest, many of practical value in medicine, and in the arts.

There however is one new instrument of investigation—a method of analysis now employed by the chemist—so entirely *new* in its nature and its power, that it must not be passed over. I refer to the *spectrum* analysis, developed principally by the researches of Bunsen and Kirchoff. This has already revealed to us four new elements, thallium and indium, and two alkaline metals resembling potassium. In this analytical method each element points its own peculiar image in the spectrum by which it may be distinguished from any other, and by which it may also be recognized in any state of combination or mixture, for it depends on the one sole property which the transforming influence of the chemical force fails to reach. And finally chemical analysis by this method attains a delicacy almost inconceivable. A 6–1,000,000 grain of lithium and 1–180,000,000 of one grain of sodium can be detected—a platinum wire which is perfectly clean and shows no trace of this metal, exposed to the air a short time, discloses on its surface the sodium in the atmosphere. More than this—the spectroscope has not only small fingers and can pick up infinitesmal traces,—it has long arms and reaches out through the celestial spaces 190,000,000 miles to the sun, and tells us what gases make its wide, surging, flaming atmosphere—nay, it stretches out to the broader distances beyond, and brings back an account of the composition of the fixed stars and nebulæ.

It is time to proceed to an examination of the progress made, during the same space of time, in the practical application of these elemental sciences to the prolongation of human existence, or the saving of human life.

The Practice and Principles of medicine first claim attention. By this is meant those grand truths and doctrines which have been ascertained and established with more or less precision by the continued observation of attentive minds throughout the entire progress of medicine as a science. Hippocrates first added philosophy and reasoning to experience, and introduced those discussions which finally led to the overthrow of empiricism. Since then, although the medical profession has uniformly conjoined the results both of reasoning and experience, each of these two methods has had its especial advocates. Even at this day, you often meet individuals who complacently call themselves "*practical men*" and sneer at all modern advances in pathology and diagnosis. On the other hand, we frequently encounter persons who attribute too much importance to theory, and regard with feelings of contempt, him, whom they denominate a "routine practitioner." Observers have not been sufficiently guarded against the danger on the one hand, of becoming mere machine-like routinists, on the other, of skepticism in relation to the efficacy of all remedial measures. The cultivators of medicine have ever been in haste to render the science *exact*,—when an individual has brought forward what he conceived to be a law, or fact, he has tried to make it applicable to all vital phenomena, and thus erected a new theory. In this way arose the doctrine of the fluidists, the solidists, the vitalists and many others. Indeed, mischievous as this effort has been in its consequences, it is not unreasonable to hope that medicine is destined to advance, and that the day will arrive when a Newton, a Galileo, or Lavoisier will arise whose genius will furnish *our* science with its primitive fact, and stamp upon it the character of precision and exactitude.

The greatest improvement which we have to chronicle, as occurring during the last forty years, in the practice of medicine, consists in the broader and more rational views now taken of disease. Physiology and pathology guide in the diagnosis and treatment, instead of a pre-conceived theory. It would now be impossible for any one to lead a large number of the better portion of the profession into the adoption of a system so partial and unsatisfactory in its foundation, as Broussaisism or *gastro-enterite* which prevailed to a great extent at the commencement of the period under consideration. No practitioner of the present day deems it necessary to bleed simply because the patient has pneumonia. No intelligent physician would be sustained in giving Tartar Emetic or Turpeths Mineral when croup was diagnosed, at least until its peculiar character was ascertained. He would feel bound in these as in many other instances which might be cited, to analyze the symptoms, ascertain the pathological condition, and after a rational examination of the case, he might conclude to pursue the course formerly adopted, but most likely, guided by the light of modern discoveries, he would find this heroic medication worse than useless. It is true the practice of medicine is now less simple, than formerly, when but to name the disease, pointed out the routine of treatment.

Forty years ago Avenbrugger and Laennec had pointed out the use of the stethoscope and described many of the intrathoraic sounds, but a fraction only of the profession were familiar with their writings, and few relied upon Physical exploration as a guide in diagnosis. To-day by these signs or sounds, every well educated practitioner expects to map out disease and determine the tissues affected, the progress it has already made, and ascertain with considerable accuracy its probable duration and termination. Forty years ago, the Microscope was used by a few progressive men more to gratify scientific curiosity then to aid in diagnosis and therapeutics. To-day, this instrument is found on the table of every well informed practitioner, and constantly called in requisition as a guide to his conclusions. The knowledge of the relation of local lesions to constitutional conditions has been greatly extended. The attention of the profession has been recently directed to fouled drinking water as a cause of cholera and typhoid fever,—to dampness of soil as cause of phthisis—thus possessing the public of the mode of preventing both of these diseases. It is only recently that locomoter, ataxy, leucocythemia, cerebro-spinal meningitis, embolism fibrinous deposits in the valves of the heart, parasitic diseases of the skin, Addison's and Bright's diseases, have been eliminated from other maladies with which they were previously confounded, their nature studied, and their therapeutics to some extent established.

It is also chiefly within the same period that diseases of the mind have received that consideration which is now conceded them. Within a short time Griesinger, Gray and Hammond have urged the importance of teaching psychological medicine clinically, as a part of the curriculum in the regular college course, in the just hope of dispelling the ignorance which prevails on this subject.

The improvement of our acquaintance with fluid blood poisons, as in septicæmia, pyæmia, etc., is but recent. The chemical tests of the urine and other excretions were seldom resorted to at the commencement of the epoch now under consideration. To-day, who would be warranted in writing a prescription, for a patient in any doubtful case, without first subjecting the urine to a careful analysis? Diseases, which it is impossible to detect without, are now often, by the addition of a few drops of acid, or a salt of copper and potash, or by the application of heat, diagnosed with certainty, the accompanying symptoms explained, and an intelligent therapeutical course adopted.

Among the aids in diagnosis, as well as treatment, which have been introduced and made available within the last few years, may be enumerated in addition to the stethoscope and microscope, already named, the ophthalmoscope, which has effected a revolution in the treatment of diseases of the eye, the laryngoscope scarcely less useful in exposing to view the organs of speech—the larynx and the pharynx. To these may be added the otoscope, the edoscope and the sphymograph. These are all now in the hands of active men who are daily enlarging their sphere of usefulness. Thermometry of the human body in fevers, and in a great variety of other diseases, now found so important in diagnosis, is of recent introduction. Perhaps in nothing has so great a change been wrought in the practice of medicine as in the improved hygienic and sanitary measures which appertain to the arrangement of the sick room and the management of the patient. Free ventilation and moderate temperature are now substituted for the confined and heated air of the sick chamber. The patient is now sustained by an abundance of easily assimilated nutriment during the progress of protracted disease, whilst formerly he was drenched with the thinnest soups, or with tisans only, and often, at the same time, in obedience to some hobby of the practitioner, subjected to the most powerful "antiphlogistic" remedies. Now, tonics and stimulants often come in to our aid in carrying a patient safely through a self-limited disease, whilst formerly their use was forbidden from an unfounded apprehension of "increasing the fever" until after the subsidence of all active symptoms. Did time permit, we could greatly add to this catalogue of changes in the practice of our profession, during the last two-score years, but we must pass on to a consideration of the claims of Surgery, which has peculiar attractions to the progressive young man, has by no means been outstripped in the race.

This brilliant department has been revolutionized within the last few years. Says Gross, "the contrast between the surgery of former times and that of the present day, forms one of the the brightest pages in the history of human progress and human achievement. Redeemed and purified by the genius of modern discovery, it is no longer a handi-craft but a science and an art. Thus improved and perfected surgery, can no longer be separated from medicine, and no surgeon of to-day can undertake the practice of his profession with credit to himself or benefit to his fellow creatures without being deeply grounded in a knowledge of the great doctrines of disease and its cure.

To enumerate all the changes which have fallen under my own observation would be impossible. Simply glancing at the works of Bell and Dorsey—brilliant representatives of that day, and then at those of Gross and Holmes—standards of to-day will abundantly illustrate the change in this department. The introduction of anæsthetics has been an unspeakable improvement in operative surgery. It has extended the field of operations and annihilated suffering. It has increased safety from lessened shock. The metallic suture, another American improvement, has rendered many operations successful, which formerly were not considered feasible.

By the combined effects of anæsthesia and the suture of his own invention, Dr. Marion Sims has demonstrated the curability of those most loathsome affections, the fistulous openings in the pelvic viscera, which were formerly abandoned as hopelessly incurable. Orthopedic surgery and the more rational treatment of all curvatures and deformities does not, in the present improved method resorted to, ante-date the commencement of our historic range. The removal of deformity by transplantation, has been immensely extended and improved within the same period. The treatment of aneurism by compression, coagulation, etc., etc., falls within the same time. Acupressure was first introduced to the attention of the surgeon by Sir Y. Simpson, in 1860. The ecraseur with all its various modifications and applications has but recently been brought into general use. The antiseptic treatment of wounds is also a recent improvement. The same may be said of the application of atomized fluids to diseased surfaces. Conservatism has, in modern surgery, asserted its perogatives, and many operations formerly believed necessary are now deferred or omitted altogether—adding much to the preservation of members of the human body, and the safety of life. Exsections, and resections of bones now often save an extremity, which formerly would without hesitation, have been doomed to amputation, and the saving of the periosteum in modern times, often reproduces a lost bone. Nor is conservatism confined to this narrow sphere—it is manifested throughout the whole range of surgery. Perhaps in no department of surgery has improvement been more manifest than in ophthalmology. In 1861 Helmholtz discovered the ophthalmoscope, and commenced a new era in the diagnosis and treatment of diseases of the eye. The development of the present theories of refraction and accommodation has greatly perfected the science. Since this discovery, and the development of the facts therewith associated, a large group of cases has been found amenable to treatment which formerly puzzled the oculist and was deemed incurable. Since the discovery of Helmholtz nearly all the diseases now known to affect the retina and interior of the globe have been investigated and their character and treatment pointed out. Iridectomy, for the cure of glaucoma, was first proposed by Von Graefe in 1856, to which may be added the suggestions of Bowman of London, since made, upon the same subject. The linear extraction of cataract also by Von Graefe as well as other greatly improved methods by other surgeons, may be mentioned, as among the modern improvements in this department. The method of removing the globe for the cure of sympathetic ophthalmia was first devised by Bonnet and O'Farrell in 1841, independently of each other. Iridodesis in 1857, by Critchett, syndectomy by Furnari, in 1862, the modern method of treating closure of the ducts and many other improvements, deserve our attention, did time permit.

The advances made in aural surgery are scarcely less than those made in ophthalmic. The otoscope is nearly as useful in the investigation of diseases of the ear, as the ophthalmoscope in those of the eye, and its beneficial results are almost as widely acknowledged. As an aid in diagnosis, the exploring trochar must be mentioned as a modern improvement of great value. The mode of dressing wounds, the treatment of the stump after amputation, Reids, and other more philosophical modes of reducing dislocations than formerly prevailed, the modern method of simplifying dressing in the treatment of fractures, the present operation for paracentesis thoracis, the introduction of lithotripsy—these and many other subjects should be carefully considered in a complete description of improvements in modern surgery. But time fails, and we proceed to ask your attention in conclusion to the last and most important of all the practical departments.

The practice of Midwifery, rather the bestowment of help on parturient women, must have been nearly co-existent with the human species. The earliest history which we possess, the Scriptures, make frequent mention of female practitioners among the Hebrews and the Egyptians. It is worthy of remark, as we pass, that in all ages, and in every country, China perhaps alone excepted, the practice has been in the hands of females down almost to the present era. Phænarete, the mother of Socrates, was a midwife; and Plato explains the functions, and regulates the duties undertaken by these females in Greece. Indeed it would seem most probable that this department of medicine was the first of necessity which was exercised as an art; and it is quite certain that it is the last to receive the highest state of improvement. Hippocrates, although often styled the father of Obstetrics, as well as Medicine, must have been profoundly ignorant upon most subjects connected therewith, as he was utterly debarred any opportunity of obtaining information at the bed-side of the patient, where alone correct observation can be made. From his day until long after the commencement of the Christian era, midwifery made no progress. Although practiced as an art it does not aspire to the dignity of a science, until late in the sixteenth century, during the reign of Ambrose Pare. About this period Hotel Dieu appropriated a ward to lying-in-women and enabled Moriceau, profiting by these unparalleled advantages, in 1668, to publish to the world a work of unsurpassed value. Some idea may be formed of the state of the science a century since, and the amount of learning supposed requisite to its practice, when we know that the great Smellie kept suspended from his office, in London, a paper sign with these words: "Mid-wifery taught for five shillings." About the middle of the last century Hunter made his appearance in this field, and with the introduction of male practitioners which now took place, and a variety of other circumstances which contributed to forward progress in this department, it has now attained a degree of certainty and of perfection not inferior to either of her elder sisters. Under the combined influences of civilization and religion, obstetric practice gradually advanced, triumphing over every obstacle, even that which seemed most formidable, the false delicacy which opposed male attendance, and which precluded careful personal observation. Shippen and Bard, and Tenant, and Boyd introduced the practice and teaching of this department into this country about a century since, and from that period, midwifery has been taught in every Medical College in the United States.

The impetus given to the study of this subject, by these great men of the last century, has gone on increasing in geometrical ratio. The introduction of the metroscope, the sound, and other modern instruments, has wonderfully developed its cognate science—gynecology, which scarcely claimed existence when I commenced the practice of medicine. Its progress is to be noticed in every journal of medicine, and its more recent history must be familiar to all. Within the last two-score years it has ascended in the scale of importance with giant strides, until it now may be said to occupy the vantage ground. Accoucheurs and gynecologists are no longer considered, as in the days of Shippen, as belonging to, or an appendage of medicine or surgery, but are assuming their just position. It is now established, that to make successful practitioners they must unite all the pathological and therapeutical attainments of the physician to the skill and energy of the surgeon, hence, more correct to assign them, at this day, a position indicating that they are a class superior to either, combining all that is meritorious in both.

When can the physician require more accuracy in investigation or more judgment in prescribing, than he who assumes the treatment of a case of child-bed fever? When can the surgeon be placed in circumstances of greater responsibility than the gynecologist who undertakes the removal of large ovarian tumours; or the accoucheur who hazards two lives on the success of a single operation?

A great change in the estimate of the comparative importance of the different departments has been effected within the period so often referred to.

The diseases of females have claimed the intellect of the greatest disciples of the profession of modern times. Of the Harveys, the Hunters, the Dewees, and more recently the literature of this department has been enriched by Simpson and Bennett and Churchill and Velpeau and Dubois and Sims and Wells and Baker Brown and Elliot and Thomas and Quackenbush and Byford and Hodge and Meigs and Bedford and a host of others. And I hesitate not to assert that more progress has been made in the diagnosis, pathology, and treatment of the affections of the organs concerned in the generative functions of the female during the last forty years, than in any other department of medical practice within the same period. Thousands of victims who formerly would have descended bed-ridden to their graves, almost without sympathy, because their diseases were not recognized and appreciated, are, by the aid of modern science, restored to their families and to society.

But I cannot detain you even to enumerate the various improvements in this interesting branch of medicine. It but remains that I beg you to concede it proved that medical science is thoroughly alive and progressive, as shown in this very imperfect resume, and to beg your pardon a thousand times for my prolixity. Finally, permit me to ask the pretending charlatan, who so wisely proclaims the stationary condition of medical science, and loudly insists that his crude ideas shall be pronounced correct because they are novel and startling, to ask of him I say, to point out one single discovery or improvement in all this long catalogue which is not a triumph for legitimate medicine.

